# Tight junctional protein family, Claudins in cancer and cancer metastasis

**DOI:** 10.3389/fonc.2025.1596460

**Published:** 2025-07-04

**Authors:** Wenxiao Ji, Xinguo Zhuang, Wen G. Jiang, Tracey A. Martin

**Affiliations:** ^1^ Division of Cancer and Genetics, Cardiff China Medical Research Collaborative, University School of Medicine, Cardiff, United Kingdom; ^2^ Center for Precision Medicine, The First Affiliated Hospital of Xiamen University, School of Medicine, Xiamen University, Xiamen, China

**Keywords:** claudin, CLDN, tight junction, blood brain barrier, metastasis

## Abstract

Claudin (CLDN) family proteins are key components of tight junctions in epithelial and endothelial cells, crucial for controlling paracellular permeability and cell-cell adhesion. Aberrant CLDNS expression is frequently observed in cancers and has been linked to tumor progression, invasion, and metastasis. Recent years have seen a rapid advance in exploring the role played by this protein family in cancer and cancer metastasis, and even chemotherapy response. This article provides a comprehensive overview of the roles of CLDNs in solid tumors, highlighting how specific CLDN members function as oncogenic drivers or tumor suppressors in different cancer types. We also discuss the potential of CLDNs as biomarkers for prognosis and therapeutic targets (e.g. CLDN18.2-targeted immunotherapy). The inclusion of updated literature (particularly post-2020) and bioinformatic analyses (TCGA/GEPIA) reveal emerging trends. Finally, we summarize common patterns of CLDN dysregulation across cancers and outline future research directions, including pan-cancer CLDN analyses and translational strategies.

## 1 Introduction

**Figure 1:**
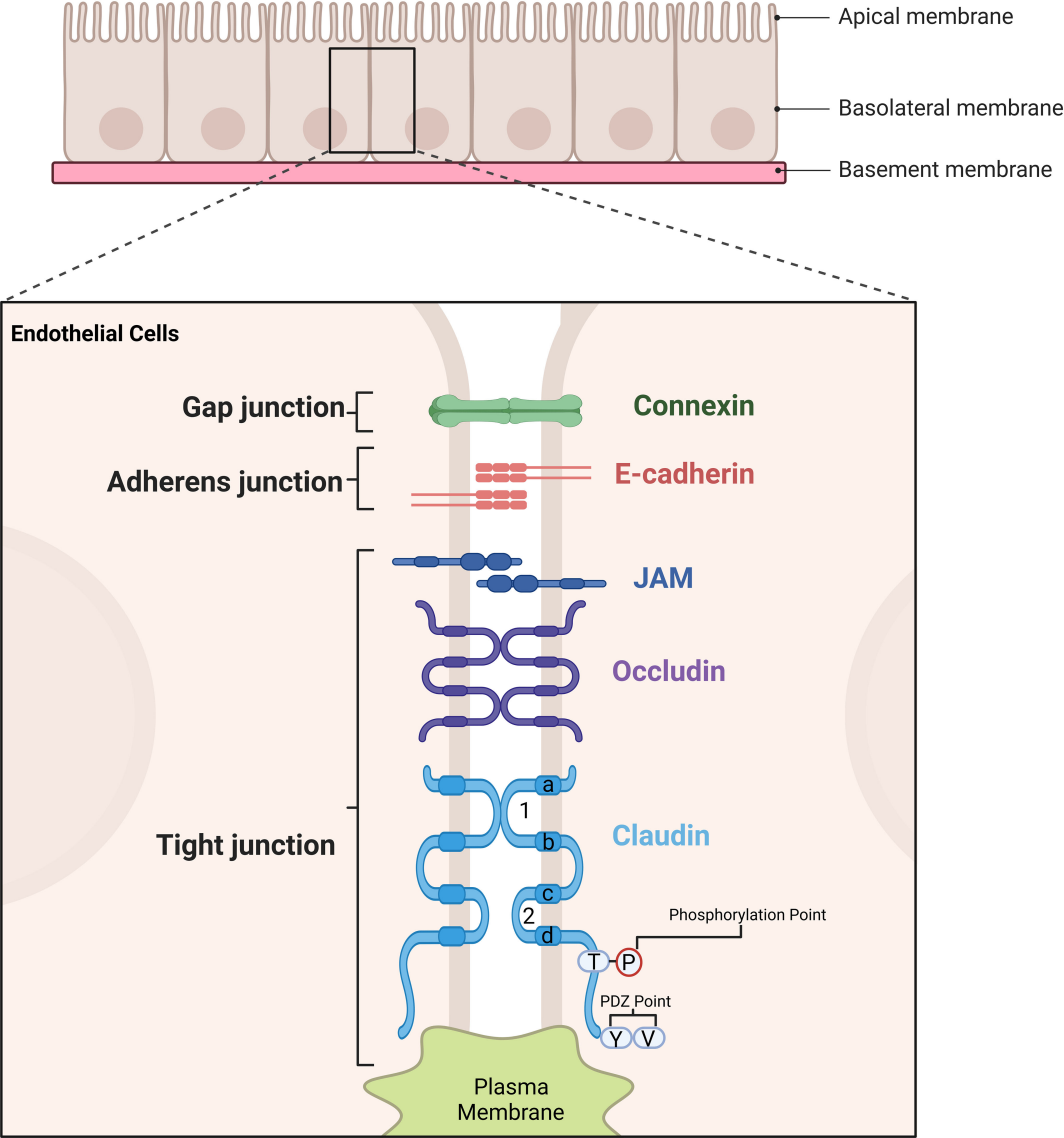
Illustration of the structural components of cell junctions in endothelial and epithelila cells. Tight junctions are formed by key proteins such as Claudins, Occludin, and Junctional Adhesion Molecule (JAM). Claudin proteins are shown with their extracellular loop domains and four transmembrane regions (labelled as a, b, c, d), which are critical for their function. The amino acids in the transmembrane regions a and d, as well as in the extracellular loop, are highly conserved, ensuring structural integrity and functional consistency. The first extracellular loop plays a pivotal role in determining the transmembrane resistance of tight junctions and the selectivity of paracellular pathways, directly influencing barrier function. Meanwhile, the tight junction structure is stabilized through interactions involving the transmembrane domains and cytoplasmic regions. These regions also contain phosphorylation points and PDZ-binding motifs, which are essential for interactions with scaffold proteins, further enhancing the stability and functionality of tight junctions. Collectively, these structural features enable tight junctions to maintain cell polarity, barrier integrity, and selective permeability between adjacent cells.

Claudins belong to a protein family that are exclusively located in the tight junctional area of cells. To date, the family has 27 members which have a molecular weight ranging between 22 to 25 kDa (1–3). Claudins 1-10, -14, -15, -17 and -19 share sequence homology and functional similarity and are often referred to as classical claudin (4). These proteins form the backbone of tight junction strands, their transmembrane and cytoplasmic domains mediate the assembly of tightly attached filaments independent of the extracellular domain (5). The simple structure diagram is shown as **Figure 1**. The extracellular loops of claudins on adjacent cells interact to seal the intercellular space, contributing to tight junction formation and selective ion permeability (5). Claudins, together with other tight junction proteins, maintain cell polarity and the barrier/fence functions of epithelia. In cancers, abnormal CLDN expression disrupts tight junction integrity, leading to loss of cell polarity and uncontrolled paracellular diffusion – changes that are hallmark features of tumor invasion and metastasis (6).

The mechanisms by which claudins influence cancer progression extend beyond structural loss of cell adhesion. Claudin proteins also interface with key signaling pathways involved in cancer. For example, CLDN dysregulation can modulate epithelial-mesenchymal transition (EMT) through interactions with EMT-regulating transcription factors (e.g., Snail, Slug, Twist) (7). Claudin-mediated signaling crosstalk with EMT pathways has been reported; for instance, CLDN1 can upregulate ZEB1 and repress E-cadherin in colon cancer cells to promote invasion (7, 8). Specific CLDN members have emerged as diagnostic and prognostic biomarkers in certain malignancies. Notably, CLDN18.2 – highly expressed in gastric and pancreatic tumors but restricted in normal tissues – has become a focus of targeted therapy development (1, 9). An overview of CLDN gene expression profiles, associated signaling mechanisms, and clinical relevance in different cancers is presented in **Table 1**. By exploring claudin structure-function relationships and their diverse roles in tumor biology, we can better understand how tight junction disruption contributes to metastasis, and how CLDNs might be leveraged as therapeutic targets. (Throughout this review, gene symbols (e.g., CLDN1) are italicized to denote the gene, while the protein is referred to as CLDN1).

**Table 1:** Comprehensive summary of claudin (CLDN) family expression, functions, and mechanisms across human cancers.

Tumor Type	CLDN	Expression	Mechanism/Effect	Sample (n)	Reference
TNBC (breast cancer)	*CLDN1*	↓ (low)	*CLDN1* loss is associated with lymph node metastasis.	174	(10)
TNBC (breast cancer)	*CLDN4*	↑ (high)	*CLDN4* upregulation enhances tumor microenvironment maintenance and stemness.	–	(11)
Breast cancer	*CLDN2*	↓	INNO-406 reduces *CLDN2* expression, suppressing liver metastasis.	–	(12)
Breast cancer	*CLDN2*	↑	High *CLDN2* expression correlates with lymph node metastasis and advanced stage.	37	(13)
Breast cancer	*CLDN6*	–	ERβ inhibits migration/invasion via *CLDN6*.	–	(14)
Breast cancer	*CLDN6*	↓	*CLDN6* loss leads to increased HIF-1α-driven metastasis.	–	(15)
Breast cancer	*CLDN8*	↓	Low *CLDN8* expression is associated with lymph node metastasis.	142	(16)
Breast cancer	*CLDN20*	↑	*CLDN20* overexpression reduces epithelial barrier resistance (TEER).	144	(17)
Lung cancer	*CLDN1*	↑	*CLDN1* upregulation induces invasion/metastasis suppressors.	67	(18)
Lung cancer	*CLDN1*	↑	*CLDN1* represses progression via the EPHB6–ERK1/2–SLUG signaling axis.	–	(19)
Lung cancer	*CLDN1*	↑	TNF-α induces *CLDN1* via the PKCδ–iPLA2–PGE2–PPARγ pathway.	–	(20)
Lung cancer	*CLDN2*	–	Spi-B disrupts junctions and promotes invasion by reconfiguring *CLDN2*.	–	(21)
Lung cancer (SqCC)	*CLDN3*	↓	*CLDN3* is downregulated in lung squamous carcinoma, correlating with progression.	–	(22)
NSCLC	*CLDN1*	↑	*CLDN1* increases cisplatin (CDDP) resistance via autophagy activation.	–	(23)
NSCLC	*CLDN3*	↑	*CLDN3* promotes tumor invasion via estrogen receptor α.	–	(24)
NSCLC	*CLDN5*	↓	High-dose bevacizumab reduces *CLDN5*, increasing tumor invasion.	–	(25)
NSCLC	*CLDN6*	↓	*CLDN6* is significantly under-expressed in NSCLC tissues.	–	(26)
Lung cancer	*CLDN7*	–	*CLDN7* co-localizes with integrin β-1 to form a complex in lung cancer cells.	–	(27)
Lung adenocarcinoma	*CLDN18.1*	↑	*CLDN18.1* attenuates malignant properties; re-expression rescues migration.	–	(28)
Gastric cancer	*CLDN1*	↑	β-catenin levels positively regulate CLDN1 expression.	–	(29)
Gastric cancer	*CLDN1*	↓	*CLDN1* depletion blocks TNF-α-induced gene expression changes.	–	(30)
Gastric cancer	*CLDN1*	↑	*CLDN1* acts as a tumor suppressor and is a direct target of RUNX3.	–	(31)
Gastric cancer	*CLDN3*	↑	High *CLDN3* contributes to initiation of intestinal-type gastric cancer.	–	(32)
Gastric cancer	*CLDN4*	↑	*CLDN4* is highly expressed in differentiated intestinal-type carcinoma.	55	(33)
Gastric cancer	*CLDN6*	↑	*CLDN6* upregulation is associated with lymph node and distant metastasis.	213	(34)
Gastric cancer	*CLDN7*	↑	*CLDN7* overexpression correlates with lymph node metastasis.	117	(35)
Gastric cancer	*CLDN8*	↓	*CLDN8* downregulation is associated with lymph node metastasis.	100	(36)
Gastric cancer	*CLDN18.2*	↑	High *CLDN18.2* expression in diffuse-type gastric carcinoma.	263	(37)
Gastric cancer	*CLDN18-ARHGAP*	–	*CLDN18-ARHGAP* fusion linked to clinical features (age, sex).	–	(38)
Gastric cancer	*CLDN18.2*	↓	*CLDN18.2* expression is decreased in peritoneal metastasis.	77	(39)
Gastric cancer	*CLDN18-ARHGAP*	–	*CLDN18-ARHGAP* fusion gene is enriched in younger-onset gastric cancer.	–	(40)
ESCC (esophageal SCC)	*CLDN1*	↑	*CLDN1* upregulation promotes proliferation and metastasis in ESCC.	–	(41)
ESCC	*CLDN4*	↓	Low *CLDN4* correlates with higher stage and lymphatic metastasis.	–	(42)
OSCC (oral SCC)	*CLDN1*	↑	*CLDN1* expression correlates positively with lymph node metastasis.	–	(43)
Tongue SCC (TSCC)	*CLDN1*	↓	Loss of cell-surface *CLDN1* promotes cell migration.	83	(44)
SACC (salivary ACC)	*CLDN7*	↑	*CLDN7* inhibits proliferation and metastasis via Wnt/β-catenin inactivation.	–	(45)
HCC (hepatocellular CA)	*CLDN1*	↑	Camptothecin increases *CLDN1*, inhibiting EMT and metastasis.	–	(46)
HCC	*CLDN1*	↑	*CLDN1* is upregulated in HCC and inversely correlates with miR-29a.	–	(47)
HCC	*CLDN3*	↓	*CLDN3* knockdown significantly suppresses metastasis in HCC cells.	–	(48)
HCC	*CLDN5*	↓	Low *CLDN5* expression correlates with poor differentiation and invasion in HCC.	51	(49)
HCC	*CLDN9*	↑	*CLDN9* activates the Tyk2/STAT3 pathway, enhancing metastatic ability.	–	(50)
HCC	*CLDN11*	↓	miR-99b directly inhibits CLDN11 expression in HCC.	–	(51)
Pancreatic ductal CA	*CLDN4*	↑	*CLDN4* upregulation correlates with tumor invasion and metastasis.	–	(52)
Pancreatic neoplasms	*CLDN18.2*	↑	High *CLDN18.2* positivity in pancreatic cancers (not limited to primaries).	130	(53)
PDAC (pancreatic CA)	*CLDN18.2*	↑	*CLDN18.2* upregulation is associated with lymph node and distant metastasis in PDAC.	192	(54)
Colorectal cancer	*CLDN1*	↑	*CLDN1* upregulation promotes colon cancer invasion and metastasis.	–	(55)
Colorectal cancer	*CLDN2*	↑	*CLDN2* upregulation increases tumorigenicity and liver metastasis.	–	(56) (57),
Colorectal cancer	*CLDN3*	↑	SCF/c-Kit/JNK/AP-1 signaling significantly upregulates CLDN3.	24	(58)
Colorectal cancer	*CLDN1/CLDN4*	↓	*CLDN1* and *CLDN4* expressions are reduced in stage IV and metastatic CRC (liver).	40	(59)
Colorectal cancer	*CLDN6*	↓	*CLDN6* is low in CRC cells; its overexpression inhibits migration and invasion.	–	(60)
Ovarian cancer	*CLDN3/CLDN4*	–	*CLDN3* and *CLDN4* sustain an epithelial phenotype; loss promotes EMT.	–	(61)
Ovarian cancer	*CLDN4*	↓	*CLDN4* suppression significantly increases cisplatin sensitivity.	43	(62)
Cervical cancer	*CLDN1*	↑	*CLDN1* induces EMT through interaction with SNAI1.	–	(63)
Cervical cancer	*CLDN8*	↑	*CLDN8* is upregulated and associated with lymph node metastasis.	72	(64)
Endometrial cancer	*CLDN6*	↑	High *CLDN6* is associated with lymph node and distant metastasis.	173	(65)
Endometrial cancer	*CLDN9*	↑	High *CLDN9* correlates with poorer prognosis (reduced survival).	173	(66)
ccRCC (kidney)	*CLDN7*	↓	*CLDN7* downregulation potentiates EMT and tumor progression in ccRCC.	144	(67)
ccRCC	*CLDN8*	↓	*CLDN8* suppresses proliferation and invasion of ccRCC cells (low in ccRCC).	148	(68)
ccRCC	*CLDN10*	↓	*CLDN10* overexpression inhibits growth and lung metastasis of ccRCC.	–	(69)
Papillary thyroid CA	*CLDN1*	↑	*CLDN1* is associated with increased risk of metastasis in PTC.	–	(70)
Mesothelioma	*CLDN4*	↑	*CLDN4* expression is specific and sensitive for metastatic carcinoma vs. mesothelioma.	49	(71)
CCAS	*CLDN4*	–	*CLDN4* suppression reduces cell migration and invasion.	–	(72)
Osteosarcoma (OS)	*CLDN8*	↓	*CLDN8* knockdown induces G1–S cell cycle arrest and apoptosis.	–	(73)
Osteosarcoma (OS)	*CLDN12*	↑	*CLDN12* promotes proliferation and migration via PI3K/Akt signaling.	34	(74)
Cutaneous SCC (cSCC)	*CLDN11*	↓	p38δ-dependent *CLDN11*; knockdown enhances cSCC invasion.	–	(75)

Arrows (↑, ↓) indicate increased or decreased expression; “–” denotes no specific change or not reported. Original reference numbers are preserved.

## 2 CLDNS and mechanisms of cancer metastasis

Invasion and metastasis are hallmarks of malignancy closely tied to patient prognosis. The loss of cell-cell adhesion is a critical step in metastasis (76). Claudins, as major tight junction components, are integral to maintaining adhesion and polarity; thus, their dysregulation can facilitate metastatic spread. When tumor cells undergo EMT, tight junctions are dismantled. Transcriptional repressors such as Snail, Slug, Twist, Zeb1, and Zeb2, which drive EMT, often lead to *E-cadherin* loss (77, 78). While E-cadherin’s role in EMT is established, claudins’ involvement is an evolving area (79, 80). Emerging evidence suggests claudins may also act as effectors of EMT in certain contexts. For example, the knockdown of *CLDN1* was shown to promote EMT and metastasis in pancreatic cancer via β-catenin signaling, indicating that maintaining CLDN1 expression can counteract EMT induction (79). Additionally, claudins can influence signaling pathways that modulate motility and invasion. For instance, the PDZ-binding motif on claudins links to scaffolding proteins (ZO-1, etc.) affecting actin dynamics and integrin signaling (81). In breast cancer, the PDZ motif of CLDNs contributes to anchorage-independent growth and adhesion to extracellular matrix components (fibronectin, collagen IV), promoting metastatic colonization (81).

Another layer of complexity is the context-dependent function of specific claudins. Some CLDNs can have opposing roles depending on the tissue or molecular environment (82, 83). This will be evident in the cancer-specific sections below. Broadly, overexpression of certain *CLDNs* in tumors can enhance proliferation, migration, and invasive capability, whereas loss of other CLDNs can remove restraints on metastasis (1). In the following sections, we detail CLDN expression patterns in various cancers and how they influence tumor behavior.

## 3 CLDNS and cancer metastasis

### 3.1 Breast cancer

Claudin proteins show highly subtype-specific dysregulation in breast cancer. A recognized *claudin-low* intrinsic subtype (often triple-negative by definition) is characterized by low expression of CLDN3, CLDN4, CLDN7 and other tight-junction molecules (84). Claudin-low tumors are enriched for epithelial–mesenchymal and stem-like features and, importantly, exhibit heavy immune and stromal cell infiltration relative to other subtypes (84). Notably, only about 36% of claudin-low tumors are triple-negative; the rest include ER-positive cases, indicating claudin-low biology transcends receptor status (84). In general, basal-like/TNBC and claudin-low cancers share poor prognosis and aggressive behavior, but claudin-low tumors tend to show higher immune infiltration (84).

Among claudins, *CLDN1* is nearly universally downregulated in TNBC. Clinical and experimental data show ~77% of TNBC lack CLDN1, and loss of CLDN1 correlates with worse survival and chemoresistance (85). Conversely, restoring CLDN1 in TNBC cells re-sensitizes them to standard chemotherapies (5-FU, paclitaxel, doxorubicin) (85). Thus, CLDN1 behaves as a metastasis suppressor and chemo-sensitizer in breast cancer: its absence promotes EMT and invasion, while its expression helps maintain epithelial adhesion and chemosensitivity (85). Existing studies have demonstrated that Stanniocalcin-2 (STC2) can regulate the protein kinase C/CLDN1 pathway to inhibit breast cancer invasion and metastasis. Hou et al. (86) confirmed that knocking out *STC2* leads to increased invasion/metastasis, implicating STC2’s regulation of CLDN1 as a suppressive axis. Meanwhile, a correlation analysis by Ma et al. (10) revealed that loss of CLDN1 protein expression in breast tumors correlates with higher rates of lymph node metastasis and worse outcomes. Specifically, breast cancer patients with absent CLDN1 had significantly poorer recurrence-free survival, and multivariate analysis showed CLDN1-negative status to be an independent risk factor for recurrence and death (10). Thus, CLDN1 appears to act as a metastasis suppressor in breast cancer, and its expression is a predictor of prognosis.

CLDN2 also promotes invasion in a context-dependent manner. Preclinical models have shown CLDN2 expression enhances breast cancer liver metastasis via heterotypic adhesion to hepatocytes (87). In TNBC and luminal subsets, high CLDN2 (and CLDN14, CLDN20) expression by TCGA analyses predicts worse survival (88), whereas CLDN2 is paradoxically associated with better distant metastasis-free survival (DMFS), possibly reflecting its complex roles (88).

Several other claudins are implicated in breast cancer progression. It is notable that claudins-3, -4, -5, and -6 can control the motility of breast cancer cells (MCF-7 and MDA-MB-415) (89). High CLDN4 expression has been associated with lymph node metastasis and enhanced cancer stemness in breast cancer (13), suggesting CLDN4 contributes to maintaining an aggressive tumor microenvironment. CLDN5 also regulates motility via cytoskeletal effectors (nWASP, ROCK); Escudero-Esparza et al. showed that elevated CLDN5 is an adverse prognostic factor in breast cancer (90). Consistently, CLDN9 is overexpressed in breast tumors, and high CLDN9 correlates with poor clinical outcomes and chemotherapy resistance (91). Growing evidence suggests that CLDN6 functions as a tumor suppressor in breast cancer through multiple mechanisms. Osanai et al (92) showed increased resistance to apoptosis after *CLDN6* knockdown in breast cancer MCF-7 cells, supporting the hypothesis that *CLDN6* downregulation could lead to breast cancer tumor formation, and suggesting that the *CLDN6* methylation phenotype may contribute to tumor formation and invasion. Supporting this, Wu et al (93) demonstrated that CLDN6 inhibits breast cancer cell invasion and metastasis via activation of the p38 MAPK signaling pathway; pharmacological inhibition of this pathway reversed CLDN6-mediated suppression of tumor aggressiveness. Furthermore, CLDN6 has been shown to mediate the anti-migratory and anti-invasive effects of estrogen receptor β (ERβ) by triggering a Beclin1-dependent autophagic cascade, linking tight junction regulation to autophagy in breast cancer suppression (14). There Under hypoxic conditions, *CLDN6* is transcriptionally upregulated by *HIF-1α*, suggesting it may act as part of a cellular adaptive response (15). However, loss of *CLDN6* disrupts this regulation and enhances *HIF-1α-*driven metastatic potential in a SUMOylation-dependent manner, further implicating CLDN6 in the control of breast cancer dissemination (15).

*CLDN8* is downregulated in breast cancer (16). Low expression of CLDN8 is associated with lymph node metastasis. Low expression of both CLDN8 and AR indicates poor prognosis, while their expression is positively correlated (16). Recent bioinformatic analyses (TCGA) further support CLDN9’s role: breast cancers express significantly higher *CLDN9* mRNA than normal tissue and high levels associated with shorter overall survival (94). Martin et al. demonstrated that overexpression of *CLDN20* in breast cancer cells decreases TER and thus increases their motility. and reduced trans-epithelial resistance (17). The other member of CLDN family, CLDN16, previously known as parceling and regulator of magnesium and calcium reabsorption (95) a key regulator of migration and aggressive characters of breast cancer cells (96). It has been shown to be highly expressed in breast tumor tissues compared with normal tissues and has a marked negative correlation with both ER and PGR in this cancer type (97). A reduced expression has been seen in node positive tumors and breast tumors from patients with poor clinical outcome (98, 99).

### 3.2 Lung cancer

Claudin dysregulation is also evident in lung cancers, though roles can differ between adenocarcinoma and squamous cell carcinoma (SqCC). In lung adenocarcinoma, overexpression of *CLDN1* has been shown to *suppress* migration and invasion of cancer cells (such as connective tissue growth factor, platelet reaction protein 1, etc.). Knocking out exogenous or endogenous CLDN1 increases the invasion and migration ability of carcinoma, leading to a shortened survival period for patients (18). Zhang et al. (100) in a clinical study involving 81 lung adenocarcinoma patients, demonstrated that CLDN1 is transcriptionally regulated by the c-Fos signaling pathway, and that its upregulation significantly inhibits tumor invasion and metastasis. High CLDN1 expression was positively associated with favorable overall survival in these patients. Mechanistically, CLDN1 appears to suppress tumor dissemination by downregulating zinc finger transcription factors through inhibition of the ERK1/2 (extracellular signal-regulated kinase) signaling pathway, thereby limiting the migratory potential of lung adenocarcinoma cells (19). However, CLDN1 also exhibits context-dependent roles in drug resistance. In non-small cell lung cancer (NSCLC), CLDN1 has been implicated in promoting cisplatin (CDDP) resistance. Specifically, it enhances autophagy in A549 cells, contributing to increased survival, proliferation, migration, and invasion. Knockdown of *CLDN1* in A549/CDDP-resistant cells reduces these malignant phenotypes and sensitizes cells to chemotherapy (23). Moreover, CLDN1 expression in lung cancer is inducible by inflammatory cytokines such as TNF-α, acting through the PKCδ–iPLA2–PGE2–PPARγ signaling axis. In this pathway, downstream mediators including iPLA2, PGE2, 15-keto PGE2, and PPARγ have all been shown to enhance CLDN1 transcription in A549 cells (20), highlighting the tight junction protein’s involvement in inflammation-driven tumor progression.

CLDN3 exhibits tumor-suppressive functions in lung squamous cell carcinoma (SqCC). In a retrospective study of 103 postoperative patients, Che et al. (101) found that CLDN3 inhibits tumor cell metastasis by modulating cell–cell adhesion through the E-cadherin/β-catenin signaling axis. Quantitative PCR and immunoblot analyses revealed that CLDN3 expression was significantly reduced in lung SqCC tissues compared to adjacent normal lung tissues. Consistently, enforced overexpression of *CLDN3* in the lung SqCC cell line H520 markedly suppressed cell migration, invasion, and epithelial–mesenchymal transition (EMT) (22). These findings highlight CLDN3 as a potential metastasis suppressor in lung SqCC. However, CLDN3’s role appears to be histology dependent. In lung adenocarcinoma, increased CLDN3 expression correlates with advanced disease stage and reduced patient survival. Moreover, *CLDN3* downregulation in SqCC has been linked to EMT activation via the Wnt signaling pathway, suggesting its loss may drive tumor progression and dissemination. Notably, CLDN3 plays an entirely opposite role in non-squamous non-small cell lung cancer (NSCLC). Ma et al. (24) demonstrated that in a panel of non-squamous NSCLC cell lines (including H460, H1792, H157, H292, and A549), CLDN3 acts as a pro-tumorigenic factor under the regulation of estrogen receptor α (ERα), promoting tumor cell invasion. This dichotomy underscores the context-specific nature of claudin function in lung cancers of differing histology.

In addition to CLDN3, other claudins are implicated in lung cancer metastasis, particularly to the brain. CLDN5, expressed in brain endothelial cells, plays a critical role in maintaining blood–brain barrier (BBB) integrity. It has been shown to modulate lung cancer brain metastasis through pathways involving Notch and STAT signaling (102). This finding echoes very well an early study which demonstrated the critical role of CLDN5 in the integrity of blood brain barrier (103). Recently, CLDN10 in cerebral endothelial cells has been found to play a similar role in controlling brain metastasis of cancer cells (91). *CLDN6* has been reported to be upregulated in most malignancies, including lung adenocarcinoma and squamous carcinoma, and acts as a pro-oncogene, promoting tumor migration and invasion (104). However, its role in non-small cell lung cancer (NSCLC) remains controversial. In contrast to its proposed oncogenic function, Wang et al. (26) found that CLDN6 was significantly more under-expressed in NSCLC tissues compared to adjacent non-tumorous tissues. In their study, low CLDN6 expression was associated with more advanced tumor stages, a higher likelihood of lymph node metastasis, decreased survival rates, and overall poorer prognosis. These findings suggest that CLDN6 may in fact function as a tumor suppressor in certain NSCLC contexts. The discrepancy between studies highlights the complex and possibly context-dependent role of CLDN6 in lung cancer. Contributing factors to these inconsistencies may include differences in patient populations, tissue sampling methods, monoclonal antibody specificity, experimental protocols, and interpretation criteria. As such, more standardized and comprehensive studies are required to clarify the prognostic and functional significance of CLDN6 in NSCLC.

Beyond CLDN6, other claudins such as CLDN9 and CLDN12 have also been implicated in lung cancer metastasis. In one study, silencing *CLDN9* expression in p-3LL lung cancer cells using siRNA significantly reduced their motility and invasive capacity *in vitro*, and suppressed metastatic potential *in vivo (* 105). Similarly, in lung squamous cell carcinoma, CLDN12 appears to promote EMT through activation of the Tyk2/STAT1 signaling pathway, suggesting a mechanistic basis for its contribution to tumor progression (106).

On the other hand, certain claudins promote lung tumor progression. Akizuki et al. (107) detected the expression levels of CLDN5, CLDN7, and CLDN18 in lung squamous cell carcinoma using semi-quantitative PCR and RT-PCR. They found that the expression levels of these CLDNs were significantly lower in lung squamous cell carcinoma compared to normal tissue. CLDN5, CLDN7, and CLDN18 were shown to inhibit the proliferation of human lung squamous cell carcinoma cells by suppressing the phosphorylation of protein kinase B. A study showed that high-dose bevacizumab likely increased tumor invasion and down-regulating CLDN5, which was down regulated by TGFβ1 (25). Conversely, low-dose bevacizumab increased CLDN5 expression by up-regulating PI3K and JNK expression. Lu et al. (27) confirmed through immuno-localization and immunoprecipitation that CLDN7 co-localizes with integrin β-1 and forms a protein complex in human lung cancer cells, which inhibits cell proliferation. Knocking out CLDN7 not only promotes tumor cell proliferation but also disrupts the localization of integrin β-1. A study on non-small cell lung cancer cell lines (HCC827) demonstrated that in various cancer cells and tissues, including lung adenocarcinoma, CLDN7 maintains the epithelial cell attachment of lung adenocarcinoma cell lines and inhibits their proliferation through its regulatory role with integrin β-1 (108). Although a few studies have demonstrated that CLDN7 inhibits cancer metastasis, it is important to note that CLDN7 is a novel transcriptional target and clinically relevant effector of PARP1 (109). In lung adenocarcinoma, PARP1 promotes invasion, drug resistance, and metastatic spread, in part by modulating CLDN7 expression. This highlights the possibility that, under certain regulatory conditions, CLDN7 could contribute to tumor aggressiveness, underscoring the context-dependent nature of claudin function.

The current lung cancer research on CLDN18 is mainly focused on LuAd. A study showed that the expression of CLDN18 by binding its 3’-untranslated regions (3’-UTR). Knockdown of *CLDN18* results in a decrease in the growth, migration, and invasion of lung adenocarcinoma cells. Although overexpression of *miR-767-3p* inhibits lung adenocarcinoma cell growth and migration, these effects can be rescued by repressing CLDN18 (28). When restored in LuAd cells with lost expression, CLDN18.1 significantly attenuates malignant adenocarcinoma characteristics, including *in vivo* xenograft tumor growth and *in vitro* cell proliferation, migration, invasion, and anchorage-independent colony formation (110). Therefore, the re-expression of CLDN18.1 may hold significant potential for the treatment of lung cancer.

### 3.3 Gastric cancer

Gastric cancer exhibits distinct CLDN expression changes often tied to its histological subtypes (intestinal vs diffuse). Claudin 1 is overexpressed in gastric cancer and is associated with tumor invasion and metastasis (29). Functional studies have demonstrated that silencing *CLDN1* in gastric cancer cells leads to a marked reduction in proliferation, migration, and invasive capacity (29). Mechanistically, CLDN1 promotes tumor progression by regulating apoptosis resistance, particularly through β-catenin-mediated pathways (29). Complementary findings from microarray analyses suggest that *CLDN1* downregulation impairs cell motility, potentially by interfering with TNF-induced gene expression programs (30). However, conflicting data exists. CHANG et al. (31) reported that CLDN1 is directly regulated by the tumor suppressor gene *RUNX3* and that its overexpression may paradoxically inhibit tumor growth *in vitro*, while knockdown accelerates proliferation. These discrepancies suggest that the prognostic or metastatic value of CLDN1 expression in gastric cancer is context-dependent and warrants further clarification through large-scale clinical studies and mechanistic research.

CLDN3 expression in gastric cancer also presents with inconsistent findings. Some studies report reduced CLDN3 levels in tumors exhibiting positive lymphatic invasion and deeper local infiltration, while others associate high CLDN3 expression with increased lymph node metastasis (32). Promoter hypermethylation has been suggested as a mechanism for *CLDN3* silencing in disseminated gastric adenocarcinoma, although the variability in methylation patterns between individuals and tissues makes it difficult to draw definitive conclusions (32).

CLDN4 protein plays an important role in gastric cancer proliferation and metastasis: a meta-analysis found that high CLDN4 correlates with more advanced disease and poor prognosis (32). Mechanistically, *CLDN4* overexpression can activate MMPs, facilitating invasion. Interestingly, some studies report contradictory effects of CLDN4 depending on context – in certain models, either upregulation or downregulation of *CLDN4* can induce EMT and metastasis (111). Interestingly, some conflicting reports suggest that *CLDN4* upregulation or downregulation may induce metastasis by promoting EMT (111). Histologically, *CLDN4* expression is commonly higher in well-differentiated intestinal-type gastric cancers and is often lost in the poorly differentiated diffuse type (112). Moreover, low expression of CLDN4 was related to lymph angiogenesis (112). Aberrant CLDN4 expression also weakens tight junction integrity, reducing intercellular adhesion and facilitating cancer cell dissemination. In contrast, high CLDN4 levels have been associated with enhanced barrier function and DNA hypomethylation in gastric cancer (113). Therapeutically, anti-CLDN4 monoclonal antibodies such as 4D3 have shown promise; Nishiguchi et al. demonstrated that 4D3 may sensitize tumor cells to chemotherapy by inducing conformational changes in tight junction structure (114).

There is no uniform conclusion on the expression of CLDN6 in gastric cancer tissues. Some studies have confirmed that CLDN6 expression in gastric cancer tissues is lower than in normal tissues using qPCR (34), while others have reported CLDN6 as highly expressed in gastric cancer tissues using genetic databases (115). Functionally, *CLDN6* knockdown suppresses gastric cancer cell proliferation and invasion, potentially through partial repression of YAP1 and its downstream transcriptional targets (116).

CLDN7 is widely expressed in normal gastric tissue, particularly in acinar cells, with its mRNA and protein both abundantly present (117). However, its role in gastric cancer progression remains unclear and somewhat contradictory. Some studies report a negative association between CLDN7 expression and clinicopathological factors such as diffuse type and lymphatic invasion (35), while others suggest that *CLDN7* overexpression promotes proliferation and EMT-driven invasion in gastric cancer cells. Analysis of patient samples revealed elevated CLDN7 levels in tumors compared to adjacent non-tumorous tissues, yet the association with nodal or distant metastasis was not statistically significant (35). Moreover, survival analysis showed that the presence of shorter OS in patients with CLDN7 than in patients without CLDN7 (118).

CLDN18, particularly the gastric-specific isoform CLDN18.2, has emerged as a promising therapeutic target. Epigenetic regulation of CLDN18 involves binding of the cAMP-response element binding protein (CREB) to its promoter in normal tissues, while hypermethylation of the central CpG island in cancer cells impedes this interaction, resulting in transcriptional silencing (119). Several studies have confirmed that *CLDN18* expression is significantly downregulated in gastric cancer relative to normal mucosa (38). CLDN18 exists as two isoforms—CLDN18.1 predominantly in lung tissue and CLDN18.2 specifically in gastric mucosa, where it is localized to the lateral membrane of epithelial cells in tight junctions (110). Importantly, CLDN18.2 is retained in a substantial proportion of both primary and metastatic gastric cancers, though its expression may be lost in peritoneal metastases while preserved in bone metastases, a finding that may reflect differential organ-specific microenvironments in accordance with the “seed and soil” hypothesis (120).

From a therapeutic perspective, CLDN18.2 clinical trials in gastric cancer have focused on IMAB362 (also known as zolbetuximab) is a chimeric antibody that selectively binds to CLDN18.2-expressing tumor cells with minimal off-target effects. In a phase I clinical trial (NCT01197885), IMAB362 was well tolerated and demonstrated encouraging activity in patients with metastatic gastroesophageal adenocarcinoma (121) .A phase IIa study using IMAB362 at 300 and 600 mg/m² in patients with refractory CLDN18.2-positive gastric cancer reported an objective response rate of 10%, a disease control rate of 30%, and a median progression-free survival (PFS) of 102 days, with only grade 1–3 adverse events such as nausea and vomiting (121). A subsequent phase II trial (NCT01630083) evaluated the combination of IMAB362 with the EOX chemotherapy regimen (epirubicin, oxaliplatin, capecitabine) in advanced gastric and gastroesophageal junction cancer. The results showed significantly prolonged PFS and OS in the combination group compared to EOX alone, without an increase in severe adverse events. Most side effects related to IMAB362, including neutropenia, anemia, and vomiting, were grade 1–2 and manageable (122). These findings support the clinical potential of combining CLDN18.2-targeted therapy with standard chemotherapy regimens for CLDN18.2-positive gastric cancer.

### 3.4 Ovarian cancer

Epithelial ovarian cancers (predominantly high-grade serous) show a characteristic upregulation of certain claudins. Most striking is CLDN3: TCGA data demonstrate that *CLDN3* mRNA levels are higher than CLDN4 or CLDN1 in serous ovarian tumors (123). Rangel et al. (124) first reported that CLDN3 and CLDN4 are consistently overexpressed across all major subtypes of epithelial ovarian cancer, while being either absent or expressed at very low levels in benign cystadenomas and normal ovarian tissues. This distinct expression pattern suggests that elevated CLDN3 and CLDN4 levels may contribute to the early steps of ovarian tumorigenesis by promoting neoplastic transformation of ovarian epithelium. Functional studies further support this hypothesis. Agarwal et al. (125) discovered that high expression of CLDN3 and CLDN4 can activate the activity of matrix metalloproteinase-2 (MMP-2), thereby enhancing the survival and invasive ability of ovarian cancer cells. In addition, Santin et al. (126) found that CLDN3 and CLDN4 were highly expressed in chemotherapy-resistant or recurrent ovarian cancer cell lines. When these cell lines were grafted into mice and treated with CPE intraperitoneal injections, the growth of tumors were inhibited and the survival time of mice was prolonged, suggesting that CLDN3 and CLDN4 may be potential new targets for the treatment of refractory ovarian cancer with great application prospects. Compared with chemotherapy-sensitive patients, the expression of CLDN4 is higher in ovarian cancer tissues from chemotherapy-resistant patients. Inhibition of CLDN4 leads to a significant increase in sensitivity to cisplatin and the accumulation of fluorescently labeled cisplatin (62). CLDN3 and CLDN4 function to sustain an epithelial phenotype and that their loss promotes EMT (61). This provides proof-of-concept that CLDN3 and CLDN4 are not only markers of ovarian cancer, but also potential therapeutic targets for refractory disease. Clinically, in ovarian cancer patients, CLDN4 expression is higher in tumors from chemotherapy-resistant cases, and silencing *CLDN4* in cell models increased cisplatin sensitivity. Thus, CLDN3 and CLDN4 play multifaceted roles in ovarian cancer, sustaining an epithelial phenotype and promoting survival, invasion, and therapy resistance.

CLDN9 has recently gained attention in ovarian and gynecologic cancers. CLDN9 is a tight junction protein normally limited in adult tissues, but it is expressed in ovarian cancer cells and shares homology with CLDN3/4, meaning it might also bind the C. perfringens enterotoxin (127). In fact, one study identified CLDN6 and CLDN9 as additional CPE receptors in ovarian cancer cell lines (127). While CLDN9’s functional role in ovarian cancer is not fully elucidated, its presence suggests it might contribute to the malignant phenotype or be exploitable for therapy similarly to CLDN3/4. More broadly, the oncofetal claudins CLDN6 and CLDN9 (normally silenced in adult tissues) are aberrantly activated in ovarian cancers (128). Their co-expression in tumors like ovarian and endometrial cancer hints at shared regulatory mechanisms that could be targeted.

In summary, ovarian cancer exhibits a pattern of claudin upregulation (CLDN3, CLDN4, CLDN7, CLDN9) that supports tumor growth, dissemination, and chemoresistance. These findings underscore the potential of claudin-based strategies in ovarian cancer: for example, CLDN3/4 targeting agents (like CPE fragments or monoclonal antibodies) and perhaps CLDN6/9-directed immunotherapies [CAR T cells against CLDN6 are already in trials for ovarian tumors (1)]. Incorporating CLDN profiling in ovarian cancer may improve prognostic stratification and identify patients eligible for such novel therapies.

### 3.5 Squamous cell carcinoma of the upper gastrointestinal tract

Oesophageal cancer is a gastrointestinal cancer with a relatively poor prognosis, which is mostly composed of squamous cell carcinoma. *CLDN1* is localized in the nucleus of oesophageal cancer cells, and its expression is upregulated in oesophageal cancer tissue, which is associated with lymph node metastasis (41). When the expression of CLDN1 is downregulated, the proliferation, invasion, and migration ability of esophageal cancer cells are significantly reduced (129). CLDN1 protein can act as a regulator of cancer cell autophagy. Rapidly growing cancer cells require autophagy to meet their high levels of energy and nutritional demands (130). In a state of starvation, the activation of AMP-activated protein kinase (AMPK) leads to increased expression of CLDN1 protein, promoting autophagy (41). In addition, CLDN1 can induce autophagy and promote oesophageal cancer cell proliferation and metastasis by activating the AMPK/STAT1/ULK1 signaling pathway (41). Low expression of CLDN4 is associated with advanced T stage, lymph node metastasis, and recurrent status (42). By establishing two stable CLDN4-silenced ESCC cell lines, CLDN4 was found to inhibit growth, colony formation, and invasion (42). Related experimental studies at Oral squamous cell Carcinoma (OSCC) have shown similar results, with CLDN1 expression was positively correlated to lymphatic metastasis in OSCC patients, which prevents Withaferin A from damaging the motility of OSCC cell (43). *CLDN8*, a squamous cell marker in the oral cavity, is downregulated in OSCC. High expression of CLDN8 is associated with a decreased overall survival rate in OSCC patients (131). However, it is worth noting that in Tongue squamous cell carcinoma (TSCC), CLDN1 on the cell surface prevents the migration of cells motility of TSCC-derived cells was increased by deficiency CLDN1 (44). In salivary adenoid cystic carcinoma (SACC), the expression of CLDN7 is decreased and significantly associated with lymph node metastasis, recurrence, and gender. CLDN7 inhibits cell proliferation and metastasis in SACC by inactivating the Wnt/β-catenin signaling pathway (45).

### 3.6 Hepatocellular carcinoma

Tight junction proteins are the main components of the tight junctions in liver cells and are involved in the formation of the blood-bile barrier in the liver. They are crucial for liver function, and defects in their integrity may lead to serious pathological and physiological consequences such as bile accumulation, cirrhosis, and even liver cancer. CLDN1 is an initiating protein for liver cancer cells, which can promote the expression of EMT-related transcription factors Slug and ZEB1 and interact with them. In cirrhosis, liver cancer, and even normal liver tissue, it induces EMT by activating the cAb1-Ras-Raf-1-ERK1/2 signaling pathway, and through this mechanism, cirrhosis transformation can progress towards malignancy (132). A study found that *CLDN1* is upregulated in HCC and negatively correlated with miR-29a expression, and miR-29a suppressed cancer growth and migration though decreasing the expression level of CLDN1 (47). According to the corresponding mechanisms, one study found that Camptothecin (CPT) reduced metastasis of Huh7 cells possibly by inhibiting EMT by upregulating the expression of *ZO-1*, *E-cadherin* and *CLDN1 (* 46). The epigenetic silencing of *CLDN14* is significantly correlated with advanced cancer stage and cancer invasiveness (133). The downregulation of *CLDN11* by miR-99 is associated with HCC metastasis (51), while the downregulation of *CLDN3* is believed to promote EMT through the Wnt-β-catenin signaling pathway. *CLDN3* downregulation occurs in 76.3% of primary HCC. Through downregulation of *GSK3B*, *CTNNB1*, *SNAI2*, and *CDH2*, *CLDN3* could significantly suppress metastasis by inactivating the Wnt/β-catenin-EMT axis in HCC cells (48). The expression of *CLDN5* in sinusoids is downregulated with the increase in liver disease or fibrosis grades. Poor differentiation and vascular-biliary invasion are significantly associated with the decrease in *CLDN5 (* 49). CLDN9 enhances the metastatic ability of liver cells by affecting the Stat3 signaling pathway through Tyk2 (50). However, more data is needed to fully understand the roles of these TJ proteins in the pathogenesis of HCC.

### 3.7 Pancreatic cancer

Pancreatic ductal adenocarcinoma (PDAC) is another aggressive malignancy where claudin abnormalities are documented. In normal pancreas, CLDN1 is expressed on ductal cell membranes, especially in well-differentiated epithelium. In PDAC, CLDN1 expression tends to be *downregulated* as tumors dedifferentiate. Reduced CLDN1 and altered localization (cytoplasmic relegalization or loss from junctions) have been associated with EMT and tumor progression (134, 135). Reduced CLDN1 and altered localization (cytoplasmic relegalization or loss from junctions) have been associated with EMT and tumor progression (134, 135). Conversely, maintaining or restoring CLDN1 expression can impede invasion/metastasis of pancreatic cancer cells (136). During the progression of pancreatic cancer, the epithelial-mesenchymal transition (EMT) plays a crucial role. As a major component of tight junctions in epithelial cells, CLDN1 not only promotes EMT when its expression is suppressed but also promotes downstream target genes FAK and Paxillin phosphorylation, further enhancing pancreatic cancer migration and invasion. Therefore, promoting the expression of CLDN1 may be an important factor in inhibiting the invasion and metastasis of pancreatic cancer cells (137, 138). This is somewhat analogous to CLDN1’s protective role in lung and breast cancers, suggesting that in certain epithelial tumors CLDN1 helps preserve junctional integrity and epithelial phenotypes.

CLDN4 is highly expressed in PDAC and has been implicated in its invasiveness. High CLDN4 levels in PDAC correlates with lymph node metastasis and distant metastasis. Targeting CLDN4 for therapy can increase the effectiveness and safety of drug treatment (52). In patients with pancreatic ductal adenocarcinoma (PDAC), the expression of CLDN18.2 often increases and is significantly associated with lymph node metastasis, distant metastasis, neural invasion, staging, and survival rates of PDAC patients (54). In pancreatic neoplasms patients, CLDN18.2 exhibits a high positivity rate, and its expression is not confined to the primary lesion, but is also retained during metastasis (53).

### 3.8 Colon cancer

Chronic inflammation is a key driver of colorectal carcinogenesis, and CLDN1 has emerged as an important link between inflammation and cancer in the colon. CLDN1 can modulate inflammatory signaling; it regulates Notch signaling by influencing MMP-9 and p-ERK pathways, thereby affecting colon epithelial proliferation and goblet cell differentiation. High CLDN1 activity hinders goblet cell maturation and reduces mucosal defense gene expression (e.g., *MUC2*, *KLF4*, *TFF3*), which increases susceptibility to colitis and inflammation-associated tumorigenesis (139). Kim et al. (140) examined 260 colorectal cancer specimens and found that 42.7% showed complete loss of CLDN1 expression. Interestingly, loss of CLDN1 in colon tumors was significantly associated with larger tumor size, vascular invasion, deeper invasion, and higher lymph node metastasis. This seems counterintuitive given CLDN1’s pro-tumor role described elsewhere; it suggests a subset of CRCs undergo *claudin switching* where CLDN1 is lost in very advanced tumors, possibly due to EMT. However, other data indicate CLDN1 is generally pro-metastatic in CRC: patients with high CLDN1 have more aggressive disease (141), and experimentally, stable shRNA suppression of CLDN1 in highly metastatic CRC cells significantly inhibited their metastatic ability in a mouse spleen-to-liver metastasis mode (55). Taken together, CLDN1 appears to have a complex role in colon cancer – it may facilitate early invasion and metastasis, but its absence in certain contexts (late-stage tumors) further exacerbates malignancy, possibly due to complete loss of junctional control and full EMT.

CLDN2 is another promoter of colon cancer progression. Increased CLDN2 expression is observed in colon tumors and is linked to tumor growth. CLDN2 may promote carcinogenesis via the EGFR/ERK1/2 pathway (56, 142). In CLDN2-deficient CRC cells, re-expression of CLDN2 led to increased proliferation, anchorage-independent growth, and tumorigenicity (56). Moreover, recent work demonstrated that CLDN2 is functionally *required* for colorectal cancer liver metastasis: silencing *CLDN2* greatly impairs the ability of CRC cells to seed and survive in the liver (57). High CLDN2 in primary tumors also correlates with poor overall survival and shorter metastasis-free survival in CRC patients (57). These findings underscore CLDN2 as an important driver of CRC metastasis and a potential prognostic biomarker.

CLDN6 is normally absent or low in colonic epithelium but can be ectopically expressed in CRC. In one study, CLDN6 was expressed at low levels in a CRC cell line (SW1116); when CLDN6 was experimentally overexpressed, it suppressed the cells’ migratory and invasive abilities, apparently by activating the tumor-suppressive TYK2/STAT3 pathway (60). This suggests CLDN6 may have a *tumor-suppressor* function in colorectal cancer, consistent with it being a developmental protein normally silenced in adult tissue.

CLDN7 is a well-documented tumor suppressor in colon cancer. Normal colon mucosa has robust CLDN7 at cell junctions, but CLDN7 is frequently lost in CRC, especially in metastatic lesions (141). One report noted *CLDN7* downregulation in ~80% of colon adenocarcinomas. The loss of CLDN7 contributes to a more mesenchymal, stem-like cancer cell phenotype (143). Bhat et al. showed that *CLDN7* loss in CRC leads to upregulation of *Sox9* and activation of Wnt/β-catenin signaling, conferring cancer stem cell properties and chemoresistance (144). Conversely, maintaining CLDN7 expression can limit tumor aggressiveness and has been associated with better response to cisplatin (141). Thus, *CLDN7* downregulation marks a subset of CRC with poor prognosis. Notably, a gene signature of high CLDN1 and low CLDN7 was able to identify high-risk, chemoresistant CRC patients (144). This underscores how the balance of different claudins (CLDN1 vs CLDN7) influences tumor behavior: CLDN1 up and CLDN7 down defines a particularly aggressive colorectal cancer phenotype.

### 3.9 Cervical cancer

Soble et al. (145) found that basal cells in normal cervical squamous epithelium expressed *CLDN2*, while spinous and granular layer cells expressed *CLDN1*, *CLDN4*, and *CLDN7*. At *CIN2-3*, *CLDN1*, *CLDN2*, *CLDN4*, and *CLDN7* were highly expressed throughout the entire epithelial layer. However, *in situ* or invasive cancer tissues showed significant downregulation of *CLDN1*, *CLDN2*, *CLDN4*, and *CLDN7* compared to CIN. The authors suggest that the upregulation or dysregulation of claudins in cervical epithelium is an early molecular event in cervical cancer, promoting the carcinogenesis of squamous epithelial cells. Late-stage low expression of claudins is related to the malignancy and invasion of cancer tissues, possibly promoting the infiltration and dissemination of cancer tissues. The significant changes in CLDN1 during this evolution process may aid in differential diagnosis. Increased expression of CLDN1 in cervical cancer cells has been linked to increased resistance to apoptosis and invasive ability (63). CLDN1 interacts with SNAI1 to induce EMT (63).

Beyond squamous cell carcinoma, claudins play roles in cervical adenocarcinoma as well. A recent study found *CLDN8* is upregulated in cervical cancer and associated with lymph node metastasis, marking it as a potential pro-metastatic factor (146). Similarly, high expression of CLDN6 is associated with lymph node metastasis and lymphatic vessel infiltration in cervical adenocarcinoma (146). These oncofetal proteins (CLDN6, normally not expressed in adult cervix) may contribute to the aggressive behavior of certain cervical cancers. CLDN9 has also been implicated, as it is one of the few genes whose expression can predict patient survival in endometrial and cervical cancers (94). While not extensively studied in cervix, CLDN9’s association with lymphatic invasion in cervical tumors has been noted, paralleling its poor prognostic impact in endometrial cancer.

In summary, cervical cancer progression is marked by a dynamic modulation of claudins – with early lesions showing claudin overexpression and late-stage cancers showing claudin loss (for *CLDN1,2,4,7*), alongside sustained or newfound expression of other claudins (*CLDN6,8,9*) that promote metastasis. This pattern reflects the requirement of early tumors to breach the epithelial barrier (via claudin dysregulation) and the need for invasive cancers to abandon tight junctions entirely. Therapeutically, the high expression of certain claudins in preinvasive lesions suggests they might be targets for preventing progression, whereas the reliance of invasive cancer cells on claudin loss suggests therapies could aim to re-introduce or mimic claudin function to restore cell-cell adhesion and reduce invasion.

### 3.10 Endometrial cancer

Endometrial cancer originates from glandular epithelium and is histologically classified into type I and type II. Type I is oestrogen-related and mainly develops from endometrial hyperplasia. In contrast, type II tumors—most commonly endometrial serous carcinomas—are estrogen-independent and characterized by high-grade histology, early metastasis, and poor clinical outcomes. Sobel et al. (147) identified significant differential expression patterns of CLDN1 and CLDN2 between these two subtypes. Specifically, CLDN1 was highly expressed in type II tumors and minimally expressed in type I, while CLDN2 demonstrated the inverse pattern. These findings suggest that CLDN1 and CLDN2 may serve as molecular markers for distinguishing between the two histological types and further reinforce the binary model of endometrial carcinogenesis.Pan et al. (148) provided additional insights by showing that CLDN3 and CLDN4 expression levels progressively increase along the pathological continuum from normal endometrium to atypical hyperplasia and ultimately to endometrial carcinoma. Their elevated expression was also associated with deep myometrial invasion, implying that CLDN3 and CLDN4 may contribute to the invasive and proliferative properties of endometrial tumors.Building upon these observations,Santin et al. (149) demonstrated that type II endometrial cancer cells with high CLDN3 and CLDN4 expression could be effectively targeted using intraperitoneal administration of Clostridium perfringens enterotoxin (CPE) in murine xenograft models. Treatment significantly prolonged the survival of tumor-bearing mice, suggesting that CLDN3 and CLDN4 may represent promising therapeutic targets for the management of aggressive, treatment-resistant type II endometrial cancers.

CLDN6 is another oncofetal protein aberrantly expressed in endometrial cancer. Normally silent in adult endometrium, CLDN6 was found to be highly expressed in a subset of endometrial carcinomas, and high CLDN6 significantly associated with advanced FIGO stage (III/IV), lymph vascular invasion, positive lymph nodes, and distant metastasis (65). Zhang et al. reported *CLDN6* overexpression in ~37% of endometrial cancers, correlating with poorer outcomes (104). CLDN6-high tumors tend to be aggressive, and CLDN6 was identified as an independent prognostic factor for reduced overall and disease-specific survival in endometrial cancer (104). Such findings nominate CLDN6 as both a prognostic biomarker and a potential therapeutic target for aggressive endometrial cancer (efforts to target CLDN6 with vaccines or CAR-T cells are underway in ovarian cancer, which could extend to endometrial cancer given CLDN6’s similar ectopic expression).

CLDN9 has recently been established as a clinically significant marker in endometrial cancer. Huang et al. (2022) found that 17.3% of endometrial carcinomas exhibit high CLDN9 protein expression, and these patients had markedly worse 5-year disease-specific survival (62.8%) compared to those with low CLDN9 (87.8%) (66). Multivariate analysis confirmed high CLDN9 as an independent predictor of poor prognosis (hazard ratio ~5) (66). Moreover, CLDN9 expression strongly correlated with CLDN6 expression in endometrial tumors (66). Patients whose tumors co-overexpressed CLDN6 and CLDN9 had an especially dismal outcome (~30% 5-year survival) (66). This pairing underscores a possible cooperative effect of oncofetal claudins. The conclusion from that study was that aberrant CLDN9 expression is a powerful indicator of poor prognosis in endometrial cancer and could be used alongside CLDN6 to identify high-risk patients (66). Given CLDN9’s role in other cancers (e.g., promoting metastasis in lung models) (94), its prognostic value in endometrial cancer aligns with a broader oncogenic function.

CLDN7 plays a contrasting role in endometrial cancer. As in colon and lung, CLDN7 appears to act as a tumor suppressor in the endometrium. Li et al. reported that high CLDN7 expression is associated with favorable features, whereas low CLDN7 correlates with advanced stage and lower differentiation (150). In *in vitro* experiments, endometrial cancer cells with low CLDN7 were more proliferative and invasive. Restoration of *CLDN7* in these cells significantly inhibited their growth and invasion, whereas *CLDN7* knockdown in CLDN7-high cells increased aggressiveness (150). Therefore, loss of CLDN7 confers a metastatic advantage in endometrial carcinoma. Interestingly, a recent TCGA-based analysis found that low *CLDN7* mRNA was enriched in high-mutational-burden endometrial cancers and linked to worse survival in certain patient subsets (151). This reinforces CLDN7’s importance as a brake on tumor progression.

Clinically, claudin profiling may soon inform EC management. For example, a CLDN6-targeted antibody–drug conjugate (ADC) has shown preclinical efficacy in CLDN6+ ovarian and endometrial tumors (152). The unique cancer-selective expression of CLDN6 (absent in normal uterus (152)) makes it an ideal ADC target, and early phase trials are underway. In summary, claudin dysregulation in endometrial cancer is tied to subtype, invasion, and outcome: high CLDN6/CLDN9 confers poor survival (66, 152), and may also reflect an immune-cold microenvironment analogous to ovarian tumors. Integrating CLDN expressions (with other markers) could therefore improve prognosis and guide novel therapies for endometrial carcinoma.

### 3.11 Renal cancer

Claudin expression in renal cell carcinoma (RCC) varies by subtype. Christopher et al. (54) investigated CLDN7 in various renal tumors and found striking differences: CLDN7 was completely absent (0% positive) in clear cell RCC (ccRCC), the most common and aggressive subtype, whereas it was expressed in 67% of chromophobe RCC, 28% of papillary RCC, and 26% of oncocytomas. Therefore, they suggested that CLDN7 could be a potential histological marker to differentiate between renal chromophobe carcinoma and eosinophilic granuloma (153). Yoo Duk Choi et al. also used immunohistochemistry and found that CLDN7 was expressed in 3.4% of renal clear cell carcinoma, 34.5% of renal papillary adenocarcinoma, and 95% of renal chromophobe carcinoma (154). In a study on the expression of CLDN7 in benign and malignant renal tumors, Lin Li et al. found that CLDN7 was expressed in 100% of renal chromophobe carcinoma, 90% of renal papillary cell carcinoma, 7% of renal clear cell carcinoma, and 45% of eosinophilic granuloma (155). In recent years, research on ccRCC has made further progress. The expression of CLDN7 is low in ccRCC, and the loss of CLDN7 enhances EMT and cancer progression (67). CLDN8 inhibits proliferation, migration, and invasion of 786-O ccRCC cells via the epithelial-mesenchymal transition and AKT pathway. The mRNA and protein expression levels of *CLDN8* are significantly decreased in ccRCC (68). Overexpression of *CLDN10* inhibits the growth and lung metastasis of ccRCC and promotes cell apoptosis in an orthotopic model. *CLDN10* overexpression upregulates the acetylation and expression levels of ATP5O (ATP synthase subunit O, mitochondrial), resulting in mitochondrial dysfunction (69). Overall, studies on claudins for the kidney are more focused on renal physiological functions, while more data are still needed for renal cancer studies.

### 3.12 Other carcinomas

CLDN1 has been linked to a higher likelihood of metastasis in papillary thyroid carcinoma (PTC) (70). The expression of CLDN4 has been shown to be both sensitive and specific in detecting metastatic carcinomas, while excluding mesothelial proliferations (71). Reduced expression of CLDN4 may indicate a progression towards cellular disorientation and invasion in salivary gland carcinomas (156). In cholangial carcinoma (CCAS), increased expression of CLDN4 was observed in hyperplastic/dysplastic biliary epithelia and cholangiocarcinoma (CCA), indicating a potential role in early carcinogenesis. While suppressing CLDN4 did not affect cell proliferation in CCA cell lines, it did lead to a significant reduction in cell migration and invasion (72). In prostatic adenocarcinoma elevated expression of CLDN4 is correlated with high tumor grade, lymph vascular invasion, and positive lymph node metastasis (157). In Osteosarcoma. The knockdown of *CLDN8* in U2OS cells resulted in the inhibition of G1-S transition and a significant proapoptotic effect (73). Overexpression of *CLDN12* was observed in OS cells, where it was predominantly localized in the cytoplasm. CLDN12 was found to promote cell proliferation and migration through activation of the PI3K/Akt signaling pathway in these cells (74). The expression of CLDN11 in cutaneous squamous cell carcinoma (cSCC) cells was found to be regulated by the activity of p38δ MAPK. Knockdown of CLDN11 resulted in an increased invasion of cSCC cells (75).

## 4 Discussion

Across diverse cancer types, certain overarching patterns can be discerned in how claudin proteins contribute to malignancy. On the one hand, abnormal claudin expression disrupts epithelial and endothelial barrier architecture, which can impair normal tissue function (as seen in leaky blood-brain barrier in brain metastases) and enable tumor cells to more easily escape their tissue confines. On the other hand, claudin dysregulation is highly tissue-specific – different CLDN family members are perturbed in different cancers, reflecting the unique requirements and environments of each tumor. For example, *CLDN3* and *CLDN4* are upregulated in ovarian, endometrial, and pancreatic cancers (where they may confer growth and survival advantages), whereas *CLDN5* and *CLDN7* are commonly downregulated in lungs, colon, and head/neck cancers (consistent with their role in maintaining a less invasive epithelial state). The upregulation or loss of claudins leads to abnormal tight junctions that diminish cell polarity and adhesion, ultimately promoting invasive behavior of tumor cells. As epithelial cells transition to a mesenchymal, motile phenotype, they often relinquish claudin expression – this loss of tight junction components like claudins is a key step in EMT and correlates with increased invasiveness. In many cancers (oral, breast, colon, etc.), claudins are indeed among the effectors modulated during EMT. However, the precise involvement of each claudin in EMT appears to depend on context, and in some cases, it remains to be fully confirmed.

A notable emerging concept is the context-dependent dual role of certain claudins. As summarized in **Table 1**, claudins like CLDN7 can act as tumor suppressors in one tissue but oncogenes in another (143). The reasons for this duality are an important subject for further research. Possible explanations include differences in the complement of binding partners and signaling pathways in each tissue. For instance, CLDN7 interacts with integrins and tetraspanins (like CD81) – the downstream effect of these interactions could vary between, say, colon epithelium and ovarian epithelium. Additionally, claudin gene regulation differs by tissue: CLDN7 is repressed by the transcription factor Sox9 in colon cancer cells (158), but in ovarian cancer CLDN7 can be silenced by DNA hypermethylation or post-translational phosphorylation (158). Such regulatory divergences might underline why a claudin is lost in one cancer but not another. Pan-cancer bioinformatic analyses (using TCGA and similar databases) are invaluable for deciphering these patterns. Indeed, recent integrative studies have identified tissue-specific claudin expression signatures. For example, a *pan-cancer analysis of CLDN6* showed it is minimally expressed in most normal adult tissues but activated in several cancers (germ cell tumors, gynecologic cancers), correlating with worse outcomes (104). In uterine cancers, CLDN6 was significantly associated with patient age, stage, and survival (104), reinforcing its role as a context-specific oncogene. Likewise, analysis of TCGA endometrial cancer data highlighted CLDN9 as one of few genes predictive of patient survival (94), a finding validated by immunohistochemical studies (66). Such insights, gleaned from large datasets, underscore how *in silico* approaches can pinpoint claudin family members of clinical interest.

Considering the clinical and translational prospects, claudins present both opportunities and challenges. Certain claudins have already proven to be effective therapeutic targets. CLDN18.2 is the prime example: its consistent expression on the surface of many gastric and pancreatic cancer cells (and restriction from most normal tissues) has enabled the development of monoclonal antibodies and antibody-drug conjugates that specifically attack CLDN18.2-positive tumor cells (1). Zolbetuximab, an anti-CLDN18.2 antibody, has shown improved survival in a Phase III trial for advanced gastric cancer, validating this approach. CLDN6 is another compelling target – being an oncofetal antigen, it is targeted by CAR T-cell therapies currently in early-phase trials (for ovarian cancer and other CLDN6+ solid tumors) (1). Preclinical studies have also yielded antibodies against CLDN4 (to deliver toxins to pancreatic and ovarian cancer cells) and CLDN3. Furthermore, neutralizing antibodies and antibody–drug conjugates have been developed against CLDN1, CLDN3, CLDN4, CLDN6, and CLDN18.2 (1), and some have entered clinical trials (notably anti-CLDN6 and anti-CLDN18.2 therapies). There is also interest in CLDN4 and CLDN9 as markers of drug resistance; for instance, CLDN9 upregulation in breast cancer was associated with chemotherapy failure (94), suggesting that targeting CLDN9 or its pathway could resensitize tumors to treatment.

On the diagnostic front, claudins are finding uses as biomarkers. Immunohistochemical panels including claudins can help classify tumor subtypes (e.g., distinguishing subtypes of renal tumors or differentiating endometrial vs. cervical adenocarcinomas). Claudin-low status in breast cancer defines a whole intrinsic subtype with therapeutic implications (claudin-low breast cancers tend to be triple-negative and may respond to immune checkpoint inhibitors due to high immune infiltration). In the realm of prognosis, high CLDN2 in colorectal cancer, high CLDN6/9 in endometrial cancer, or low CLDN7 in various cancers could inform risk stratification and follow-up intensity.

Looking ahead, future research should focus on several key areas: (1) unraveling the regulatory networks that control claudin expression in cancer (including epigenetic modifications and non-coding RNAs that target claudin transcripts), (2) exploring the functional consequences of claudin dysregulation in the tumor microenvironment (for instance, how claudin-mediated barrier defects influence immune cell infiltration or nutrient gradients in tumors), (3) further pan-cancer analyses to identify less obvious patterns of claudin alteration (for example, correlations between claudin expression and specific oncogenic mutations or virus-associated cancers), and (4) advancing claudin-targeted therapies. The latter could include not only antibodies and CAR T-cells but also small molecules that modulate claudin interactions or stability. One intriguing concept is strengthening tight junctions in carcinomas to inhibit metastasis – a challenging but potentially novel anti-metastatic strategy. Conversely, selectively opening junctions in the tumor (while keeping normal tissues intact) could enhance drug delivery to poorly perfused tumor cells.

In conclusion, abnormalities in the claudin family are a common feature of epithelial malignancies. While each cancer type has a unique “claudin signature,” a unifying theme is that claudin dysregulation – be it gain or loss – often correlates with invasion and metastasis across cancer types. By continuing to explore the “claudin code” of cancers, we can gain new insights into tumor biology and uncover innovative approaches for diagnosis and treatment. The evidence compiled in this review, especially with the addition of recent findings and bioinformatic data, provides a strong rationale for considering claudins as both biomarkers and targets in the ongoing fight against cancer. Future studies and clinical trials will determine best to exploit these tight junction components to improve patient outcomes.
